# Dynamics of Different Buffer Systems in Slurries Based on Time and Temperature of Storage and Their Visualization by a New Mathematical Tool

**DOI:** 10.3390/ani10040724

**Published:** 2020-04-21

**Authors:** Veronika Overmeyer, Felix Holtkamp, Joachim Clemens, Wolfgang Büscher, Manfred Trimborn

**Affiliations:** 1 Institute of Agricultural Engineering, University of Bonn, 53115 Bonn, Germany; buescher@uni-bonn.de (W.B.); m.trimborn@uni-bonn.de (M.T.); 2 Institute of Crop Science and Resource Conservation, University of Bonn, 53115 Bonn, Germany; holtkamp@uni-bonn.de; 3 SF-Soepenberg GmbH, 46569 Hünxe, Germany; j.clemens@soepenberg.com

**Keywords:** titration, acidification, alkalization, buffer capacity, buffer curve, amount of acid, volume of alkaline, animal manure, slurry, waste management

## Abstract

**Simple Summary:**

Efficient slurry management is a key strategy to reduce the release of environmentally harmful gases produced by farm animals. Slurry treatments such as acidification and alkalization have proven to be promising solutions to reduce these emissions. In this context, it is crucial to understand how buffer capacities behave and may influence each other during storage under the influence of different temperatures. To realize this, we have developed and successfully verified a new mathematical tool. It allows an exact calculation and detailed visualization of the most important buffer systems found in the analyzed slurries. This knowledge can be used to optimize slurry treatments, as it allows faster, more precise and efficient timing of pH adjustment, thus, reducing the use of resources.

**Abstract:**

Slurry treatments such as acidification and alkalization have proven to be promising solutions to reduce gaseous emission produced by farm animals. The optimization of these technologies requires detailed knowledge of how and to what extent the buffer capacities in slurries will change during storage under the influence of different temperatures, as this may save resources needed to adjust a targeted pH value. Fresh slurries from dairy cows, fattening pigs and sows were collected and stored for 12 weeks under either cold (4.7 ± 1.1 °C) or warm (23.6 ± 2.1 °C) conditions to perform titrations in acidic and alkaline milieu at regular intervals. Based on these results, we successfully verified a new mathematical tool that we have developed to be able to calculate and visualize the most important buffer systems found in the analyzed slurries. Our experimental results showed a strong correlation between the degradation of the volatile fatty acid (VFA) buffer and the emergence of the carbonate buffers, i.e., the HCO_3_^−^ and the CO_3_^2−^ buffer. Furthermore, a drop in the pH value caused by enhanced microbial production of VFAs can be mitigated by the presence of the NH_3_ buffer. In conclusion, we demonstrated that the buffers cannot be considered individually but must be interpreted as a complex and interacting system.

## 1. Introduction

The massive release of greenhouse gases (GHGs) into the atmosphere by anthropogenic activities drives and aggravates climate change, leading to an increase in global average temperatures, changes in precipitation patterns and melting of the ice sheets, resulting in a rise in sea levels [1]. However, to mitigate these negative effects arising from GHG emissions, the EU has committed itself to reduce GHG emissions to at least 40% below 1990 levels by 2030 [2].

Improved manure management might have a high potential to achieve this goal, because in the EU in 2017, around 8% of total methane (CH_4_) and nitrous oxide (N_2_O) emissions and the biggest share of the total ammonia (NH_3_) emissions were caused by direct or indirect effects of slurry storage and application [3,4]. Ammonia emissions from the storage and spreading of digestate from the anaerobic digestion of energy crops are becoming increasingly important, as they were responsible for 10% of total NH_3_ emissions in Germany in 2017 [5]. Methane is a harmful GHG due to its 25 times higher global warming potential than CO_2_ and its long residence time of 12 years in the atmosphere [6]. Ammonia is considered as an indirect greenhouse gas, as it does not directly promote the greenhouse effect, but can be naturally converted to the climate-damaging gas nitrous oxide in the soil [7]. Besides, ammonia has many other negative characteristics, for instance, it reacts in the atmosphere with acidic compounds to form particulate matter (PM_2.5_) that is harmful to humans and animals. Furthermore, ammonia emissions may cause soil acidification and eutrophication of terrestrial and aquatic ecosystems by deposition [8]. Regions such as North-West France, Lombardy in Italy, the Netherlands, Denmark and Lower Saxony in Germany often suffer from high ammonia emissions due to a high density of livestock farms, which produce and store large quantities of slurry [9,10,11]. Most of these areas have been declared ‘nitrate vulnerable zones’, i.e., the EU Nitrates Directive applies, which sets a nitrogen limit for livestock slurry of 170 kg N ha^−1^ year^−1^ [12]. As a result, the amount of slurry produced often exceeds the available area on which it is allowed to use slurry as an organic fertilizer. The consequence in these regions is a massive overproduction of slurry, which in combination with inappropriate slurry management concepts can lead to a failure of the environmental objectives set by the EU.

**Figure 1:**
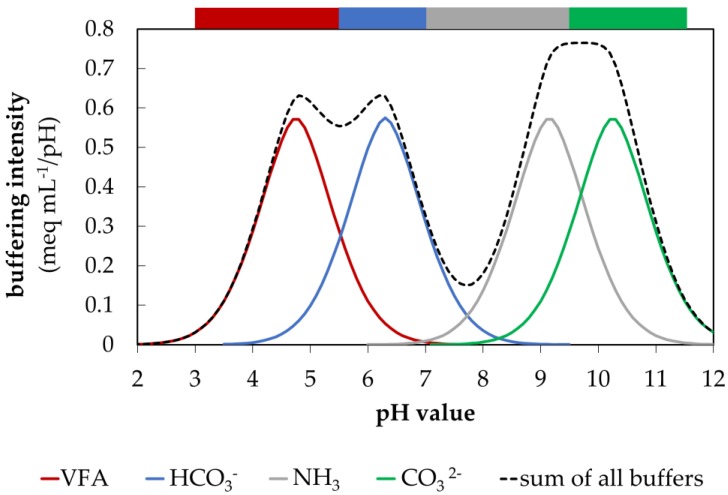
Dynamics in buffering intensity with pH for different buffers commonly found in anaerobic digesters; vertical bars indicate the boundaries between the buffer areas (modified according to [22]).

Promising approaches to avoid these problems are innovative slurry treatment technologies such as the acidification or alkalization of slurry. The equilibrium between NH_4_^+^ ⇌ NH_3_ + H^+^ is strongly pH dependent and shifts with decreasing pH value from the volatile non-ionized form NH_3_ (pKs = 9.25) towards the nonvolatile ionized form NH_4_^+^ [13,14]. Slurry which has been acidified to a pH of 5.5 may cut emissions of NH_3_ by more than 75% and emissions of CH_4_ by 94% [15]. Besides that, a 95% reduction in ammonia emissions were also found in acidified digestate [16]. In Denmark, the acidification of slurry is already a well-established method to lower emissions during the storage and application of slurry [17,18]. The alkalization of slurry is another method used to reduce emissions, but it is based on the addition of alkaline additives, that cause the precipitation of phosphorus and an increase in pH, which shifts the equilibrium towards the volatile form NH_3_ [14,19]. A so-called stripping technology enables the removal of the growing share of NH_3_ in the slurry, which is then captured and concentrated by absorption with concentrated sulphuric acid to produce ammonium-based mineral fertilizers [20,21]. It has been shown that an ammonia recovery rate of more than 90% is possible in cattle slurry at a pH of 12 [20]. The adjustment of a targeted pH value can be very challenging, as the pH value of stored slurry may fluctuate widely over time due to an increase or decrease in the buffer capacity of the four main buffer systems within slurry, which are volatile fatty acid (VFA) buffer (predominantly CH_3_COOH/CH_3_COO^−^), carbonic acid-bicarbonate buffers (H_2_CO_3_/ HCO_3_^−^ and HCO_3_^−^/CO_3_^2−^, hereafter only HCO_3_^-^ and CO_3_^2-^ buffer) and ammonia buffer (NH_4_^+^/NH_3_) (shown in Figure 1) [22,23]. Hence, the amount of acid/base must be constantly adapted to overcome the buffer capacities. These fluctuations are mainly influenced by changes in the VFA and ammonia buffer capacity [24]. The formation and degradation of the VFA buffer system depends on the ratio of anaerobic VFA-producing microorganisms that generate VFA via the decomposition of organic matter within the animal slurry and the aerobic VFA-consuming microorganisms [25]. During storage, this ratio can change considerably, which may lead to altered VFA concentrations and thus to an increase or decrease in the pH value [23]. Aeration of slurry can accelerate the decomposition process of VFA by oxidation [24]. Furthermore, the ammonia buffer system, which is mainly formed by the decomposition of urea into ammonia, could counteract the acidification of the slurry [8,23]. Both processes may cause a shift of the pH value to the alkaline state, which changes the NH_4_^+^ ⇌ NH_3_ + H^+^ equilibrium in favor of NH_3_ and thus promotes losses of NH_3_ by volatilization [24]. In addition, the decomposition of VFAs and urea produces carbonate, which can act as a buffer system in alkaline and acidic milieu and has the ability to regulate NH_3_ volatilization losses [8,26,27]. These changes in the buffer systems were expected to occur mainly in fresh slurry, whereas in older slurry or digestate microbial degradation processes are almost complete.

In order to get a better understanding of the dynamic of these different buffer systems, (I) we have designed a new mathematical tool, which can be used for a detailed description and visualization of buffer capacity curves. (II) The tool was verified by comparing the generated values with the total inorganic carbon (TIC) and total ammonia nitrogen (TAN) contents as well as with the strength of the four main buffer systems found in the slurries that were identified based on over 300 titrations. (III) Furthermore, with the help of our mathematical tool and the titrations themselves, we aimed to reveal the dynamics of the individual buffer capacities based on storage time and temperature. Thus, a more precise understanding of the microbial degradation processes in slurry can be obtained. In addition, the model enables a better prediction of the amount of acid/base required to adjust a targeted pH value, allowing this to be done faster, more precisely and at the optimal time during storage. This may help to reduce the running costs of acidification and alkalization technologies by saving resources and time.

## 2. Materials and Methods 

### 2.1. Slurry Sampling

Three fresh slurries (from fattening pigs, sows and dairy cows) not older than three days, were used for this investigation. The samplings occurred in the summer of 2019.

Two days before sampling the slurry pits of the fattening pigs (bodyweight 30–75 kg) were emptied to a small technical residual amount. The slurry sampling was done by using a sub-surface scraper system. The dairy cow slurry was taken from the walking alley of the cubicle barn. Here the slurry was collected for one hour before it was shoved off by a flap scraper. The feces and urine of high-bearing sows were collected separately for two days and then mixed.

Approx. 50 L of each slurry type was collected and divided into sample bottles necessary for the following laboratory tests (see Section 2.2).

In order not to change the microbial activity in fresh slurry the time between sampling and first analysis in the laboratory (hereafter: week 0) was kept as short as possible—between 4 (sow slurry) to 17 (dairy cow slurry) hours. Additionally, no cooling of the slurry was therefore required.

### 2.2. Storage

The influence of storage for a period of 12 weeks after removal from the slurry pits should be determined. Therefore, the titration investigations were carried out at week 0, 1, 2, 4, 6 and 12. Each slurry sample was stored in a separate 250 mL sample bottle (height: 119 mm, inner diameter: 63 mm, Low Density Polyethylene) to ensure that the samples were undisturbed during the complete storage period. The samples were stored under aerobic conditions as the lids were laid on the sample bottles (not screwed on tightly) allowing gas exchange but reducing strong evaporation losses.

In addition, the influence of the storage temperature on the buffer capacity of the slurry was investigated by comparing storage at cold (4.7 ± 1.1 °C) and warm conditions (23.6 ± 2.1 °C). Each variant consisted of three replications.

### 2.3. Analyses of the Ingredients

In weeks 0 and 8, slurry samples were analyzed by an external laboratory (AGROLAB Agrar und Umwelt GmbH, Sarstedt, Germany). The analyses included the determination of physico-chemical parameters (dry residue), macronutrients (total nitrogen (N), ammonium-nitrogen (NH_4_-N), phosphate (as P_2_O_5_), potassium (as K_2_O), magnesium (as MgO), calcium (as CaO), sulfur (S)), micronutrients (copper (Cu), zinc (Zn)) and the main volatile fatty acids (acetic acid, propionic acid, butyric acid, iso-butyric acid, valeric acid, iso-valeric acid, n-caproic acid). The acetic acid equivalent was calculated from the volatile fatty acids.

Additionally, the total ammonia nitrogen (TAN = NH_3_ + NH_4_^+^) was determined by the Quantofix-N-Volumeter [28,29,30]. As a reagent, the mixture of sodium hypochlorite and sodium hydroxide described by Klasse [30] was used. The measurement of the bicarbonate buffer expressed in total inorganic carbon (TIC = CO_2_ + HCO_3_^−^ + CO_3_^2−^) was carried out according to the methods of Clemens and Seufert [31] and Hecht [32].

### 2.4. Titration

The first slurry sample was titrated with 0.5 M HCl from the initial pH to 2.5, followed by a titration with 0.5 M NaOH from pH 2.5 to 12. The second sample was titrated from the initial pH to 12 by adding 0.5 M NaOH. A titrator (‘TitroLine 7000’, SI Analytics^®^, Mainz, Germany) with an ‘InLab Max Pro-ISM’ pH sensor (Mettler Toledo, Ohio, USA) was used for determination of pH value and temperature. For the titration process, 50 g slurry was diluted with 50 g deionized water, allowing the sample to be moved sufficiently with a magnetic stirrer. The buffer effect of distilled water was not considered due to its low ion content.

All titrations were performed via dynamic titration with a maximum step size of 0.5 mL. During the titration process, the current pH value, the amount of titrant and the temperature were automatically recorded. The pH sensor was calibrated according to manufacturer instructions. All titration experiments were performed at 24.7 ± 2.6 °C.

### 2.5. Calculations of the Titrations and New Determination Model for Buffer Capacity

**Figure 2:**
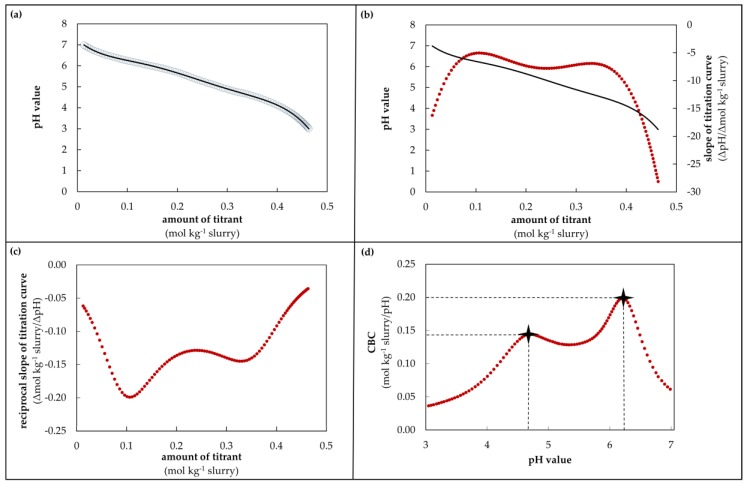
Procedure of the determination model for buffer capacity; (**a**) pH value in dependency of the amount of titrant and polynomial regression line; (**b**) polynomial regression line and derived titration curve which stands for the slope of titration curve; (**c**) reciprocal derived titration curve in dependency of amount of titrant; (**d**) current buffer capacity in dependency of pH value.

The buffer capacity was determined from the amount of titrant (HCl or NaOH) during titration. The current buffer capacity (CBC) is the amount of acid or base required to change the pH by one unit at a specific pH value. The new mathematical tool (called: determination model for buffer capacity) to calculate this CBC proceeds in several steps (Figure 2).

Firstly, the amount of titrant (mol kg^−1^ slurry) and pH value were plotted (Figure 2a). Then, the regression line (polynomial 6th degree) was calculated and mapped. In this equation, the amount of titrant is defined as the argument and the pH value is the value of the function (**a**, black line) (called: titration curve). The slope of the titration curve is equal to the first derivation of this curve. Therefore, the equation of the titration curve was derived (Figure 2b, red dots). The slope of the titration curve in dependency of the amount of titrant is outputted as ΔpH/Δmol kg^−1^ slurry. To determine the CBC, the reciprocal slope of the titration curve was formed (Figure 2c). The CBC refers to the amount of titrant per kg slurry needed to change the pH value by one unit. For visualization, the CBC (mol kg^−1^ slurry/pH) was plotted in dependency of the pH value (Figure 2d). The resulting graph represents the buffer capacity curve. The calculated maximum CBC (max. CBC) and the pH value of the maximum CBC are also exemplarily shown in Figure 2d (symbol). Furthermore, Equation (1) shows the calculation of the CBC for acidification and alkalization in a short form.

(1) $$\mathrm{CBC}=\left|\frac{1}{\mathrm{slope}\;\mathrm{in}\;\mathrm{the}\;\mathrm{respective}\;\mathrm{pH}\;\mathrm{value}}\right|$$

In order to determine the polynomial regression line needed to calculate exact CBC values, only specific value ranges of the titration data were chosen. The titration data during acidification from pH 7.0 to 3.0 and during alkalization from pH 7.8 to 11.5 were used. If the alkaline pH range began with a value higher than 7.8, the nearest value to 7.8 was chosen as the starting point. For the alkalization after acidification (pH 2.5 to 12.0), titration values from 7.0 to 11.5 were chosen and analyzed. The CBC during acidification is shown in mol H^+^ kg^−1^ slurry/pH, whereas for alkalization the CBC is given in mol OH^−^ kg^−1^ slurry/pH. In the following study, only mol kg^−1^ slurry/pH is used.

The calculation of the CBC using the model for determining the buffer capacity was carried out with the R Studio software (Version 1.0.153). However, the calculation of the regression line can also be done with other mathematical software, e.g., Microsoft Excel or Mathematica (Wolfram Research).

### 2.6. Statistical Analysis

Erroneous titrations influencing the CBC and the position of the buffers were excluded. These titrations could be easily identified as the titration curves showed strong irregularities caused by an accumulation of organic material on the pH sensor or by retention of the titrant when entering the solution due to excessive foaming.

Statistical analysis were done using IBM^®^ SPSS^®^ Statistics, Version 25. The values represent mean values and are given with standard errors of mean (SEM) either in brackets or in vertical bars, except for temperature (mean value ± standard deviation). The number of considered values is indicated with *n*. Correlation analyses were performed using the Pearson correlation coefficient (r) at a significance level of *p* < 0.05. The graphical presentation of the correlation was performed by creating a linear regression line. One-way analysis of variance (ANOVA) was performed at a significance level of 0.05 to describe the differences in pH values and the amount of acid used for the different weeks. Subsequently, the Tukey’s Honestly Significance Difference (HSD) was used, if appropriate. In the absence of variance homogeneity, the Games-Howell test was chosen with significance level of 0.05 to indicate statistical significance.

## 3. Results

### 3.1. Slurry Characterization

The three types of slurry were analyzed in terms of their physico-chemical parameters, nutrient and volatile fatty acid contents (Table 1). The dry residue content decreases for all slurries (except coldly stored dairy cow slurry) over the storage period. The decrease is higher in warmly stored slurry. Dry residue is lowest in sow slurry compared to the other types of slurry. This is also indicated by the low P_2_O_5_, K_2_O and VFA contents in the sow slurry. Instead, the NH_4_-N and TAN contents in this slurry are higher. After eight weeks of storage, the NH_4_-N contents were similar to week 0 irrespective of the storage temperature and slurry type. The highest acetic acid equivalent and thus the highest VFA concentration were detected in the fattening pig slurry. Acetic acid accounts for the greatest share of the acetic acid equivalent in all slurries. During storage, the variations in VFA and TIC contents showed no clear pattern between all three types of slurry. Further results of the slurry characteristics are shown in the Appendix A (Table A1).

### 3.2. Verification of the Determination Model for Buffer Capacity

For the evaluation of the titrations, a model to determine the buffer capacity was developed, which allows the determination and visualization of buffer capacities based on titration curves. The verification of this new mathematical tool should reveal if the calculated values (maximum CBC, pH value of the maximum CBC) are reliable. Therefore, the pH of the maximum CBC calculated with the new mathematical tool was compared with the slurry temperature at the time of measurement. In addition, the calculated CBCs were compared with the TIC, TAN and amount of acid used for a defined pH range.

**Figure 3:**
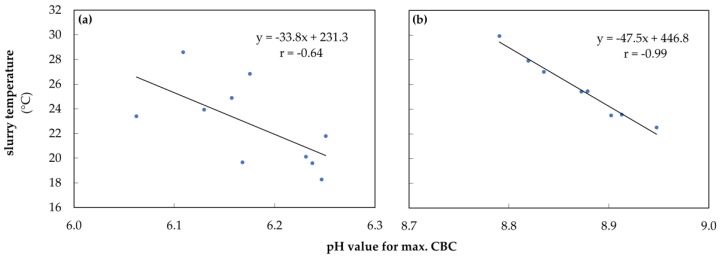
(**a**) Temperature of dairy cow slurry depending on the pH value for maximum CBC in HCO_3_^−^ buffer during acidification; (**b**) Temperature of sow slurry depending on the pH value for maximum CBC in NH_3_ buffer during alkalization from pH 2.5 to 12.0.

The first step, in verifying the model used to determine the buffer capacity was to correlate the sample temperatures with the pH values corresponding to the maximum CBCs of the HCO_3_^−^ buffer in dairy cow slurry during acidification (Figure 3a) and of the NH_3_ buffer in sow slurry during alkalization (Figure 3b). Both, the carbonate and the ammonia buffer are sensitive to temperature changes, thus the pH value for maximum CBC increases at lower temperatures and decreases at higher temperatures. Nevertheless, the ammonia buffer reacted more homogeneously to temperature changes, while the carbonate showed stronger fluctuations. This effect was checked for significance by using a correlation analysis that revealed for the carbonate buffer r = −0.64 (*p* < 0.05) and for ammonia buffer r = −0.99 (*p* < 0.001).

**Figure 4:**
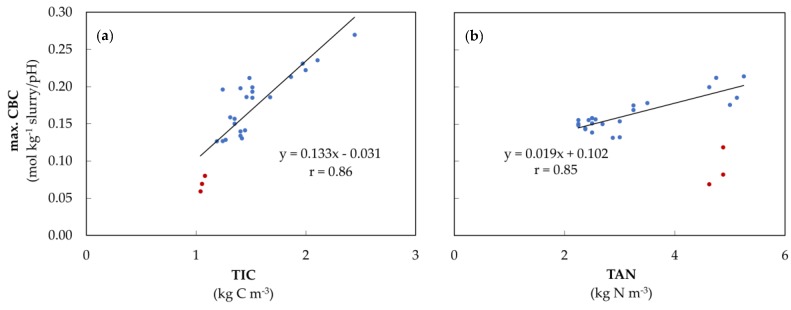
(**a**) Maximum CBC during acidification in HCO_3_^−^ buffer depending on the TIC content of the different slurries (sow slurry in red dots) in week 0 and 8 (n = 26); (**b**) Maximum CBC during titration from pH value 2.5 to 11.5 in the NH_3_ buffer depending on the TAN content of the different slurries in week 0 and 8, sow slurry of week 0 (red dots) was excluded from the calculation of the regression line here (n = 23).

The measured TIC contents and the maximum CBC values in the pH range between 6.0 and 6.5 (HCO_3_^−^ buffer) varied greatly in the analyzed slurries (Figure 4a). The maximum CBC differed between 0.06 and 0.27 mol kg^−1^ slurry/pH. The TIC content was between 1.04 and 2.44 kg C m^−3^. In week 0, the sow slurry had low TIC levels, which were also indicated by the low maximum CBC values (red dots). Besides that, a strong correlation between these parameters was observed (r = 0.86, *p* < 0.01).

After the acidification of slurry to pH 2.5, titrations from pH 2.5 to 11.5 were performed, allowing the maximum CBC of the ammonia buffer to be shown with less influence of the carbonate buffer because the previous acidification eliminated the potentially interfering carbonate buffer (Figure 4b). Furthermore, in week 0, the sow slurry was found to be exceptional, as the maximum CBC values were lower than that of the other samples, although the TAN contents were within the same range. The correlation between the TAN content and the maximum CBC of the ammonia buffer was strong with r = 0.85 (*p* < 0.01) (excluding sow slurry in week 0).

**Figure 5:**
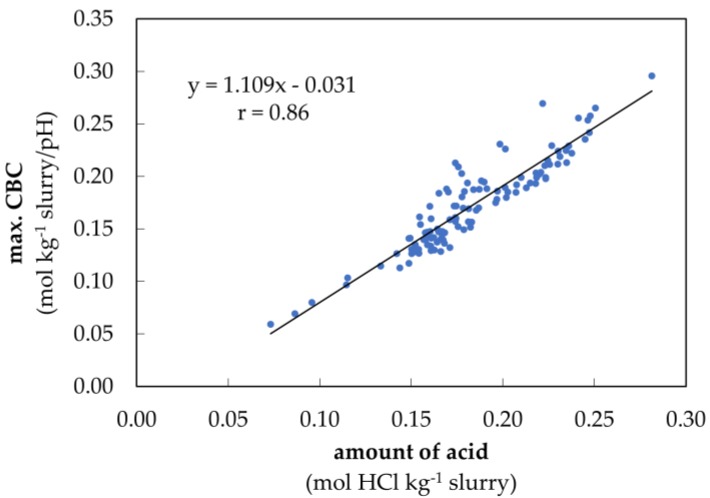
Maximum CBC during acidification in the HCO_3_^−^ buffer depending on the amount of acid from pH value 7.0 to 5.5 of the fattening pig, dairy cows and sow slurry (n = 115).

In order to reduce the pH value from 7.0 to 5.5, between 0.07 to 0.28 mol HCl kg^−1^ slurry was used. The maximum CBC in this range was between 0.06 and 0.30 mol kg^−1^ slurry/pH (Figure 5).

Moreover, it could be shown that the amount of acid in the pH range 7.0 to 5.5 is directly proportional to the maximum CBC measured within the HCO_3_^−^ buffer range. This results in a strong correlation value of r = 0.93 (*p* < 0.001).

**Figure 6:**
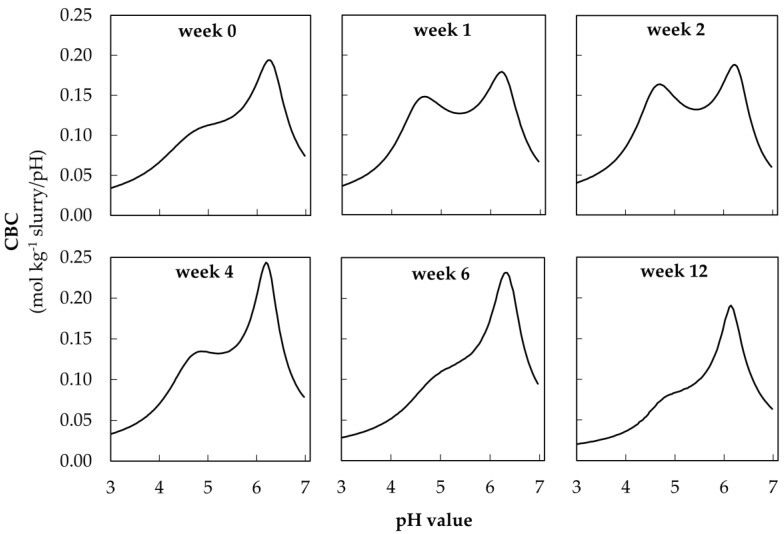
Visualization of CBC depending on pH value in fattening pig slurry stored under warm conditions (23.6 ± 2.1 °C) from week 0 to 12.

**Figure A1:**
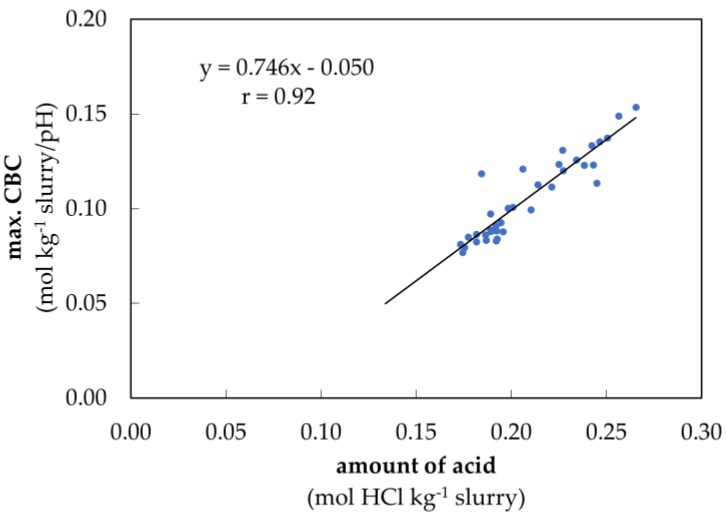
Maximum CBC during acidification in the VFA buffer depending on the amount of acid from pH value 5.5 to 3.0 of the dairy cow slurry (n = 36).

In order to compare the amount of acid in the pH range of 5.5 to 3.0 with the maximum CBC of the VFA buffer, only the maximum CBC of dairy cow slurry could be used. A correlation of r = 0.92 (*p* < 0.001) between the amount of acid and the maximum CBC was observed (Figure A1). For the other two types of slurry, the maximum CBC of the VFA buffer could not always be differentiated from the carbonate buffer. This becomes clear by the visualization of the buffer curves during the storage period in warmly stored fattening pig slurry in week 0, 6 and 12 (Figure 6).

### 3.3. Visualization of CBC during Acidification

The visualization of the buffer curves allows a graphical representation of the change in each buffer system over 12 weeks of storage. These buffer curves are shown as an example of the acidification of warmly stored fattening pig slurry (Figure 6).

In the pH range between 4.0 and 4.5, the peaks indicate the maximum of the VFA buffer, which has been defined as the maximum CBC value in this pH range. In this interval, the maximum CBC increased from week 0 up to 0.16 (0.01) mol kg^−1^ slurry/pH in week 2. Then, the VFA buffer was reduced during storage until week 12. The maximum CBC could not be exactly quantified in weeks 0, 6, and 12, because there was no local maximum (see Section 3.2). In the pH range between 6.0 and 6.5 (HCO_3_^−^ buffer) the CBC increased from 0.19 (0.01) to 0.24 (0.01) mol kg^−1^ slurry/pH after week 2 and finally decreased again to 0.19 (0.02) mol kg^−1^ slurry/pH until week 12.

### 3.4. Initial pH Value and Amount of Acid for Titration during Storage at Different Temperatures

**Figure 7:**
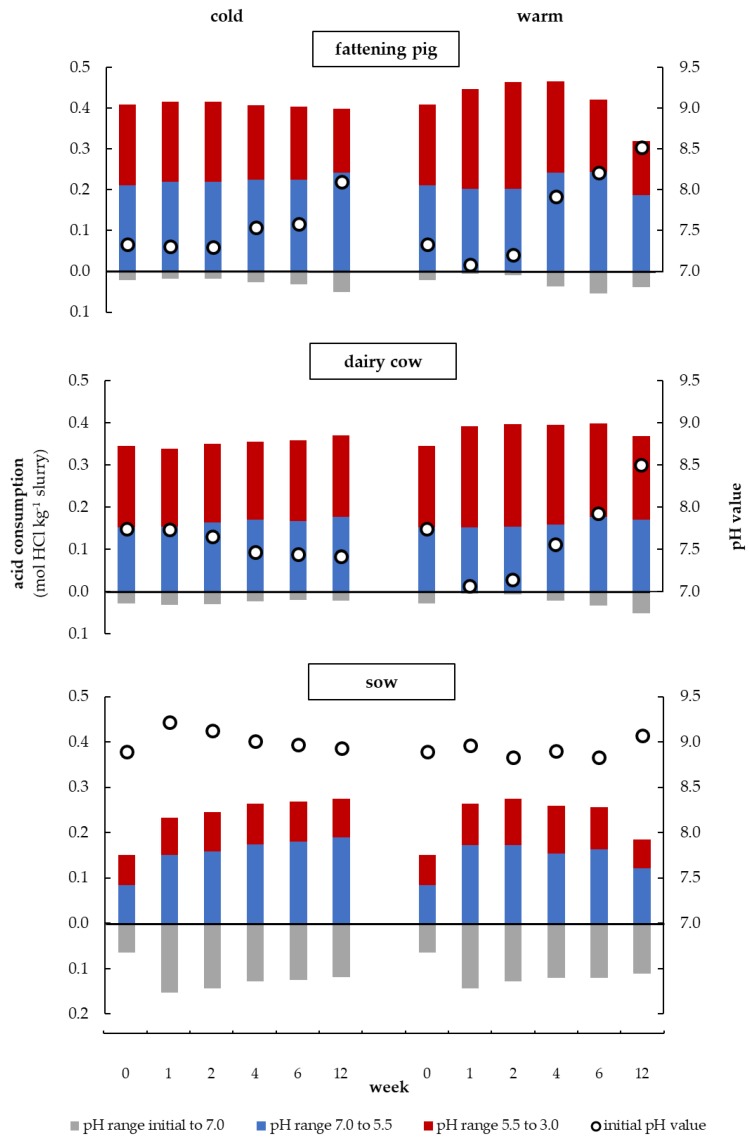
Initial pH value of fattening pig, dairy cow and sow slurry as well as the amount of acid under cold (4.7 ± 1.1 °C) and warm (23.6 ± 2.1 °C) storage conditions in the pH range initial to 7.0, 7.0 to 5.5 and 5.5 to 3.0 over a storage period of 12 weeks; for better visualization, the amount of acid in the pH range initial to 7.0 is shown below the black line.

As shown in Figure 5 and Figure 6, the maximum CBC correlates very closely with the amount of acid required to adjust the pH values in the respective pH ranges in which the buffers are located. Since the maximum CBC could not always be determined with the mathematical tool, the dynamics of the VFA and the carbonate buffer were determined in Figure 7 based on the amount of acid in the pH ranges 5.5 to 3.0 (red) and 7.0 to 5.5 (blue), respectively. The grey column below the black line represents the amount of acid needed to lower the initial pH value to 7.0. This was done to minimize the influence of the changing initial pH and the ammonia buffer on the amount of acid used in the acidic milieu. Additionally, the initial pH values are plotted in this figure. The measured parameters are shown for all three types of slurry and both storage temperatures. The individual values and significant differences are shown in Table A2.

**Figure 8:**
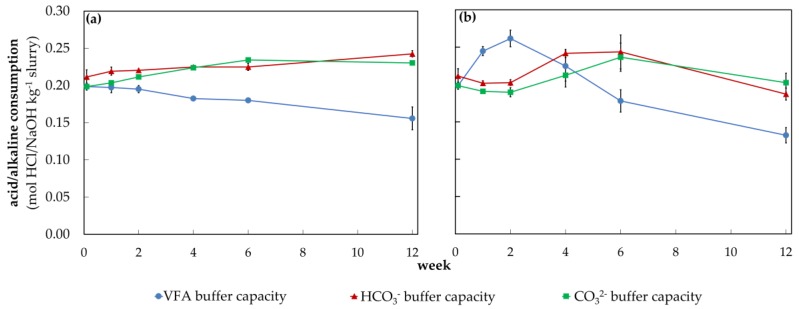
Evolution of the acid/base amount for (**a**) coldly stored (4.7 ± 1.1 °C) and (**b**) warmly stored (23.6 ± 2.1 °C) fattening pig slurry shown over 12 weeks to visualize the dynamics of the VFA (pH range 5.5 to 3.0), HCO_3_^−^ (pH range 7.0 to 5.5) and CO_3_^2−^ (pH range 9.5 to 11.5) buffer systems, vertical bars represent standard errors (n = 3).

The dynamics of the VFA and carbonate buffer during the warm storage of the fattening pig slurry as already described in Figure 6 in Section 3.3 can also be seen for the amount of acid which is shown in Figure 7 (‘warm’, ‘fattening pig’). The amount of acid from 5.5 to 3.0 (VFA buffer) increased considerably from week 0 to 2, while the amount of acid between 7.0 and 5.5 (HCO_3_^−^ buffer) remained constant during this period. Additionally, Figure 8b shows in detail the dynamics and interaction of these buffers with the CO_3_^2−^ buffer. Thus, it could be seen that during the first week the amount of base from 9.5 to 11.5 (CO_3_^2−^ buffer) remained constant as well. In the following four weeks, a close interaction between the VFA buffer and the carbonate buffer became clear, as the VFA buffer capacity rapidly decreased and the carbonate buffer rose to a peak value for HCO_3_^−^ and CO_3_^2−^ concentrations.

**Figure A2:**
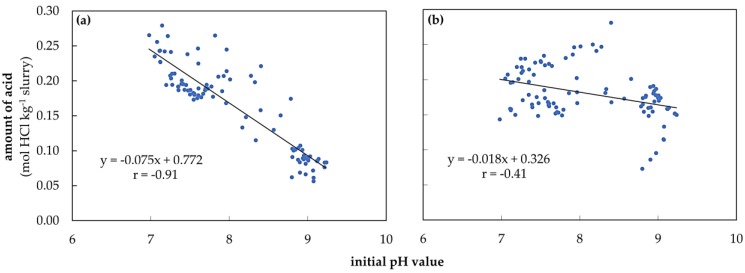
Amount of acid in pH range 5.5 to 3.0 (VFA buffer) (**a**) and in pH range 7.0 to 5.5 (HCO_3_^−^ buffer) (**b**) in dependency of initial pH value (n = 105).

Hereafter, the VFA and the carbonate buffer slowly but steadily decreased (Figure 7, ‘warm’, ‘fattening pig’). Besides that, the two carbonate buffers showed only minor differences in their curve progressions and so did they in their buffer capacities dynamics during the entire storage period (Figure 8b). The initial pH value of the warmly stored fattening pig slurry developed contrarily to the VFA buffer. This pH value initially decreased from week 0 to 1 and then increased during the entire storage period (Figure 7, ‘warm’, ‘fattening pig’). Moreover, there is a significant dependency on the initial pH value of the VFA buffer (r = 0.91, *p* < 0.001). The carbonate buffer has a lower influence on the initial pH value (r = 0.41, *p* < 0.001) compared to the VFA buffer (see Figure A2). The warmly stored dairy cow slurry showed similar dynamics of the VFA buffer, the carbonate buffer, and the initial pH value as those found in the fattening pig slurry (Figure 7, ‘warm’, ‘dairy cow’). However, in the case of dairy cow slurry, the VFA buffer was reduced from week 6 instead of week 4 (in fattening pig slurry).

Both, the coldly stored fattening pig and the dairy cow slurry showed a clear delay in the development of the buffers. In addition, the VFA buffer in the coldly stored fattening pig slurry did not show any fluctuations but instead a linear degradation (Figure 7, ‘cold’, ‘fattening pig’ and Figure 8a). Furthermore, analogies between VFA buffer degradation and carbonate buffer formation were also observed in coldly stored fattening pig slurry, as the carbonate puffer increased in a similar ratio to what the VFA buffer decreased. It is noticeable that the initial pH value of the fattening pig slurry tends to increase, whereas in the dairy cow slurry it tends to decrease.

In the sow slurry, the lowest VFA buffer capacities and the highest initial pH values and thus the highest amount of acid in the pH range initial to 7.0 (Figure 7, ‘sow’, grey columns) were found compared to the two other types of slurry. In the first week of storage, the coldly stored sow slurry showed a significant increase in the pH value, whereas the warm conditions had no significant influence on the pH value. At the same time, however, there was a much stronger increase in the VFA buffer in the warmly stored slurry than in the coldly stored slurry. Regardless of the storage temperature, the HCO_3_^−^ buffer approximately doubled its capacity during this period (*p* < 0.05).

## 4. Discussion

### 4.1. Verification of the Determination Model for Buffer Capacity

In order to demonstrate that the new mathematical tool can be used to calculate and visualize buffer capacity curves and their dynamics based on time and temperature of storage, the tool was verified by employing the results of seven different correlations. It was found that the model used for determining the buffer capacities in slurries is capable of detecting and plotting temperature-induced shifts of buffer range in both, the acidic and alkaline milieu (Figure 3). The carbonate buffer reacted with higher fluctuations to temperature changes than the NH_3_ buffer, which showed a very homogeneous response. This was probably due to the excessive foam formation during the acidification process and the high sensitivity of the carbonate buffer to an enhanced ion input (H^+^) [33]. The maximum CBC value also correlated significantly with the TIC and TAN values found in the slurry (Figure 4). Thus, with the help of our mathematical tool, conclusions can be drawn about the amount of carbonate and nitrogen in the slurry. Furthermore, the maximum CBC values of the carbonate buffer (Figure 5) and the VFA buffer (Figure A1) showed a significant correlation with the amount of acid required to overcome these buffers. In conclusion, the mentioned results show a reliable correlation between the data calculated with our mathematical tool and the data which we have obtained from actual measurements. The CBC can quantify the ‘ability’ of the weak acid/base at a defined pH value to resist a change in pH when strong acid or base is added, as is also shown in the study of Moosbrugger et al. [34].

However, the tool also has its limits, especially when the individual buffers overlap in their pH value ranges and are therefore no longer differentiable. This frequently was the case between NH_3_ and CO_3_^2−^ buffers. Hence, buffer capacity analyses in alkaline milieu were found to be particularly difficult. In the case of larger titration errors (see Section 2.6), the newly described model could not completely interpolate the data. Foam formation during acidification, especially with dairy cow slurry, complicated the titrations. This is due to rapid CO_2_ releases through the addition of strong acids [27]. It can be described as a mechanical buffering of slurry in comparison to, e.g., water.

In order to be able to compare the capacity of the individual buffers between the weeks not only visually but also statistically, the maximum CBC must be clearly identifiable. For example, this was possible for the VFA buffer only in the case of dairy cow slurry (Figure A1).

Georgacakis et al. [22] titrated different swine digesters with acid and lye. The titration curves were subjected to regression analysis so that this could be expressed as a general mathematical model (polynomial 3rd degree). After the derivation of this function, the buffer capacity was displayed. However, the authors restricted their study to the pH range 6.5 to 9.5 [22]. Other authors have shown the buffer capacity by expressing the inverse slope of the titration curve in the dependency of pH [35]. They visualized the buffer curve without performing an additional calculation of the maximum buffer capacity. It is also uncertain in which steps the respective gradient was calculated [35].

An advantage of the new model in this study is the calculation of the buffer capacity based on the titration curve which can be obtained without the need for more specific measurements as it was the case in other studies [36]. Christensen et al. [37] mentioned that they plotted the buffer capacity as a function of the pH value (only visualization). The focus of their study was on specific buffers (e.g., phosphate groups). Therefore, the carbonate was expelled by prior acidification and could not be considered in the presentation of the buffer capacity [37]. The carbonate buffer must be included in the acidification and alkalization of slurry. Our determination model for buffer capacity allows a detailed representation of the buffer curves over the whole pH range.

In a study to determine the pH buffer capacity in poultry litter, the titration curve was represented by a linear equation and a sigmoidal curve. After forming the reciprocal of the slope, the sigmoidal curve allowed to plot the pH buffer capacity as a function of pH in the range from 6.5 to 9.5 [38]. Costello and Sullivan [39] also calculated the buffer capacity of compost by the negative reciprocal of the slope. However, they used the slope of a linear regression, which represented the titration curve after adding different amounts of acid [39]. Therefore, the authors could only show a general but no corresponding buffer capacity for the current pH value.

The area under the curve between two pH values can be used to determine the amount of H^+^ ions that need to be added or removed to change the pH value [34]. This could be enabled by further calculations with our model.

### 4.2. Slurry Characterization, Initial pH Value and Amount of Acid during Storage at Different Temperatures

#### 4.2.1. Slurry Characterization

In fattening pig slurry, a higher VFA content was found than in dairy cow slurry. The predominant VFA in animal slurries is acetic acid (Table 1). This is similar to other studies [40,41,42]. Sommer and Sherlock [43] also reported that acetic acid represents more than two-thirds of the fatty acids. In contrast, Miller and Varel [44,45] found almost twice as many VFA in the cattle slurry compared to the fattening pig slurry. The microbial degradation of the acids does not change the ratio [43]. In this analysis, acetic acid is also the predominant fatty acid after a storage period of eight weeks (Table 1). Popovic and Jensen [41] reported that the VFA for pig slurry will decrease over the storage period (43 weeks). This is similar to our study. Nevertheless, no consistent tendency can be observed for the sow and dairy cow slurry. This might be explained by the slower degradation processes of the VFA in the dairy cow and sow slurry. Most of the VFAs in fattening pig slurry produced in the first weeks were degraded during storage, whereas a higher amount of VFA was still present in the other two types of slurry at the end of storage. Thus, an analysis of the VFA concentration at only two times cannot show the entire dynamics of the VFA buffer.

The dry residue is highest in the dairy cow slurry compared to the other two slurry types (Table 1). Cooper and Cornforth [40] also noted this observation. During storage, the dry residue of almost all slurries was reduced. These dry residue losses were also observed by other authors during the storage of solid manure [46], raw slurry and its liquid fraction irrespective of storage temperature [41]. In our study, the dry residue losses are higher in warmly stored slurry (confer [41]). They are due to the decomposition of organic matter, resulting in the transformation of carbon into methane and carbon dioxide [47]. At the end of the storage period, a decrease in the carbonate buffer was also observed in warmly stored slurries (Figure 6, Figure 7 and Figure 8). The resulting emissions depend on several factors such as storage temperature or the presence of an adapted microbial community in pre-stored slurry [48].

The reduction in NH_4_-N and N contents during storage, which was also presented by the authors of [41], could not be confirmed by this study (Table 1). The shorter storage period (8 weeks compared to 43 weeks) could probably be a reason.

#### 4.2.2. Initial pH Value

The pH value of fresh slurry is around 7.5 [25]. This could be confirmed for the fattening pig and dairy cow slurry, whereas the fresh sow slurry had an initial pH value of 8.9, which could be due to the farm-specific feeding. After 12 weeks, the pH value of almost all slurries had increased compared to the start pH value, especially during warm storage. Popovic and Jensen [41] observed a continuous pH increase in pig slurry during storage (Figure 7).

The pH value of slurry is strongly dependent on HCO_3_^−^/CO_3_^2−^ and NH_4_^+^/NH_3_ buffer systems and the amount of VFA [22,23]. Sommer and Husted [49] also reported a strong influence of the VFA content on the pH value of slurry. As the concentration of VFA increases (Figure 7, week 2), the importance of the carbonate buffer system decreases. The pH value is then mainly determined by VFA and ammonia concentration [22]. However, other authors declare that bicarbonate is the most important pH buffering compound in slurry [27]. In this study, it was found that the VFA buffer mainly influenced the pH value (Figure 7 and Figure A2). The study of Summer and Husted [50] explained that the pH value increases if the VFA content decreases at constant TIC concentration, whereas the pH value decreases if the VFA content remains constant but the TIC concentration increases due to enhanced CO_2_ production. The microbial oxidation of VFA can lead to an increase in the pH value because these acids have been metabolized [24,40,51]. This can be confirmed in the present study when considering the VFA progression and the initial pH starting from week 4 (fattening pig) and week 6 (dairy cow and sow) in Figure 7. VFAs are metabolized under aerobic conditions resulting in the production of CO_2_. The CO_2_ can be absorbed by the solution in various degrees [49] which contributes to the pool of TIC [25]. Besides that, low ionic concentrations lead to an increase in the pKs value of the carbonate buffer [33]. Possibly the different ionic strength of the different slurries influences the initial pH value. This could explain the low correlation between the carbonate buffer and the initial pH value (Figure A2).

The slurry pH of dairy cow [24] and pig slurry [42] was reported to be significantly correlated with VFA/NH_4_^+^+NH_3_. Eriksen et al. [52] demonstrated that an increase in pH value during the first week of storage was caused by the mineralization of organically bounded N [53]. This pH increase could only be observed for the very fresh sow slurry (cold: +0.33 and warm: +0.08 pH units from week 0 to week 1) (Figure 7, ‘sow’) because it still contains a high content of urea (see Section 4.3). The pH value of slurry from dairy cattle fed with a low nitrogen diet decreases from 8.15 (day 0) to 5.94 (day 56) [54]. In comparison to this study, it was found that the pH value during warm storage dropped sharply in the first week (but only to pH 7.07) and increased continuously again until week 12. On the other hand, the coldly stored dairy cow slurry showed a continuous decrease in the pH value (but not as strong as reported by Aguerre et al. [54]) (Figure 7, ‘dairy cow’).

The pH value of the warmly stored sow slurry is higher in week 12 than in week 2 (+0.24), although the total amount of acid required to obtain a pH of 3 is much lower in week 12 (−0.11 mol HCl kg^−1^ slurry) (Figure 7, ‘warm’, ‘sow’). This indicates a lower buffer capacity in week 12. The same tendency can be observed for all warmly stored slurries by comparing week 6 and 12. Consequently, high initial pH values do not necessarily require high amounts of acid to adjust a targeted low pH value, e.g., 5.5. It is the strength of the buffer capacity that mainly influences this amount.

#### 4.2.3. Amount of Acid in the VFA Buffer Range

During the first two weeks of storage under warm conditions, the amount of acid of all types of slurry increased in pH range 5.5 to 3.0 and thus the buffer capacity of the VFA buffer (Figure 7, red columns). This can be explained by the degradation of cellulose, hemicellulose, and lipids by microorganisms that generate acetic, propionic and butyric acid as degradation products [55]. The higher the concentration of easily fermentable carbohydrates, cellulose, and hemicellulose and the lower the lignin concentration in the feed is, the higher the concentration of VFA in the slurry [56]. The formation of organic acids under anaerobic conditions reduces the pH value [25]. Most of the VFA are formed during anaerobic storage of fattening pig slurry in the first weeks after excretion [44]. Therefore, the pH value is initially reduced in fresh slurry [25], similar to the results of our analysis (fattening pigs: +0.05 mol HCl kg^−1^ slurry and −0.26 pH units respectively dairy cow: +0.05 mol HCl kg^−1^ slurry and −0.67 pH units from week 0 to week 1). The amount of acid used in pH range 5.5 to 3.0 decreased with the length of the storage period in all warmly stored types of slurry, which indicates a reduction in the VFA buffer. The higher the storage temperature, the more the VFA content is reduced compared to coldly stored slurry [57]. Degradation processes take place even at a temperature of 0 °C [57]. Hence, the degradation of VFA in this study can also be assumed for the cold storage conditions (Figure 7, ‘cold’, ‘fattening pig’). Whereas other authors described a lower activity of the bacteria at cold conditions, so the VFA persists [40]. According to Sommer and Sherlock [43], the duration until the start of microbial degradation of VFA increases with descreasing temperature.

#### 4.2.4. Dynamics of the Carbonate Buffer and its Impacts on the Alkaline Milieu

In the anaerobic microbial transformation of organic matter, parts of the carbon hydrates are converted into VFA or directly into CO_2_ (aq) [25]. In a second step, VFA consuming aerobic bacteria decompose the emerged VFA into methane and CO_2_, which are then emitted during storage through naturally occurring volatilization processes [25,58]. In Section 4.2.3. we mentioned in more detail that the storage temperature has a considerable influence on the described carbon turnover, as the microbial conversion occurs more quickly at higher temperatures [57]. In this study, warm storage conditions caused stronger fluctuations and a faster decrease in the VFA and HCO_3_^−^ buffer compared to coldly stored slurry (Figure 7 and Figure 8), which emphasizes the mentioned temperature effects on slurry. In addition, the saturation volume of these gases in water decreases with increasing temperature, allowing less gas to be dissolved in the liquid phase of the slurry. Hence, faster and more intensive volatilization losses of CO_2_ occur at higher temperatures [58]. These losses of CO_2_ reduced the carbonate buffer capacity [43], which could explain the decrease in carbonate buffer capacity from week 0 to 2 and 6 to 12 in warmly stored slurry (Figure 8b). However, in the coldly stored slurry, the carbonate buffer increased continuously until week 12, indicating that this loss can be neglected at cold storage conditions (Figure 8a). Furthermore, we were able to show that the CO_2_ produced by the microbial decomposition of VFAs does not immediately emit, but rather functions as HCO_3_^−^ and CO_3_^2−^ buffer in the acidic or alkaline milieu. This was particularly shown by the fact that the rapid degradation of VFA in the warmly stored fattening pig slurry caused a rapid increase in both carbonate buffer concentrations (Figure 8b). Coldly stored slurry showed a similar pattern, in which continuous VFA degradation caused a continuous increase in the carbonate buffer (Figure 8a). Regardless of the storage temperature, both carbonate buffer curves showed minor differences, which confirms that the CO_2_ produced by VFA degradation can act as HCO_3_^−^ and CO_3_^2−^ buffer.

This leads to two particular findings: Firstly, the degradation of the VFA buffer does not contribute to a general reduction in the total buffer capacity in the acidic milieu, since the HCO_3_^−^ buffer itself is formed during the degradation of the VFA buffer and therefore increases in a similar proportion at which the VFA buffer degrades. Secondly, the degradation of the VFA buffer has a direct effect on the total buffer capacity in the alkaline milieu, due to the ability of the generated CO_2_ to act as CO_3_^2−^ buffer in the alkaline milieu. This leads to the paradox that despite the decomposition of VFAs and the resulting increase in pH, the amount of base needed to alkalize the slurry increases.

### 4.3. Degradation of Urea in Slurry

High TAN contents were detected in the sow slurry, week 0, which, however, showed low maximum CBC values of the ammonium buffer (Figure 4b). This contradicts the significant positive correlation between the TAN contents and the maximum CBC values of the ammonium buffer obtained for the remaining slurry types. The deviation observed in the sow slurry could most likely be attributed to the high urine content (dry residue = 2.5%) and the short storage time of fewer than four hours between the collection and the first titration of the slurry. Therefore, a large proportion of the urea in the urine has probably not yet been degraded, as this may take up to 20 h [59]. However, urea has no buffer capacity, but its N content is detectable as TAN by its reaction with hypochlorite in the Quantofix-N-Volumeter, leading to the observed erroneous values shown in Figure 4b. The fattening pig and dairy cow slurry had to undergo a longer storage time, which negated this effect due to over-advanced degradation processes of urea (Figure 4b). The sharp increase in the HCO_3_^−^ buffer capacity of 49% in the first week in warmly stored sow slurry supports the mentioned hypothesis (Figure 7, ‘sow’) because urea decomposes into ammonia and carbonic acids and thus has a direct influence on the NH_3_ and HCO_3_^−^/CO_3_^2−^ buffer formation [8]. In addition, the low CO_2_ and low maximum CBC values in the sow slurry (week 0) indicated that the urea in the slurry has not yet been fully degraded (Figure 4a).

As already described in Section 3.4, these buffer systems and the VFA buffer have a decisive influence on the pH value in slurries. High concentrations of NH_3_ in the slurry arising from the decomposition of urea may cause the pH value of slurry to rise. However, this effect only occurred at cold storage conditions, whereas the pH of warmly stored slurry remained unaffected (Figure 7, ‘sow’, week 1). This could be explained by the fact that warm storage caused a 12% increase in the VFA buffer compared to coldly stored slurry. The VFA buffer in the slurry stored under cold conditions was therefore not sufficient to compensate the increasing NH_3_ buffer and thus to counteract a rise in the pH value.

As a result, slurry acidification treatments that have been carried out without long storage periods may require considerably less acid to adjust to a target pH value than slurry in which urea has already been completely degraded. However, this does not provide any information on the pH stability of a target pH value and thus on the amount of acid needed to maintain it. The alkalization technologies employ a different approach, as they are aimed at removing high quantities of nitrogen in the form of NH_3_ from the slurry via stripping processes. That means, the storage period of the slurry must be based on the time it takes for the complete degradation of urea into CO_2_ and NH_3_. Furthermore, we found that buffers present in the acidic and alkaline milieu can compensate each other.

## 5. Conclusions

The new mathematical tool introduced in this study to determine the dynamics of buffer capacities in slurry has proven its effectiveness, as it is capable of calculating and simultaneously visualizing individual buffer capacity curves. Based on the long storage period of 12 weeks, the warm and cold storage conditions and the small step intervals in which the slurry was analyzed, we were able to determine and visualize the dynamics of the buffer capacities in great detail. The experimental results showed that greater changes in VFA and HCO_3_^−^ buffer concentrations occurred over the whole storage period when the slurry was stored in warm conditions. On the other hand, less strong but constant increases were found in cold storage conditions. Furthermore, a strong dependency between the buffers present in the acidic and alkaline milieu was observed during storage, indicating that buffers cannot be considered individually, but must be interpreted as a complex and interacting system. Based on these results, we can provide a recommendation for acidification technologies, stating that immediate acidification of slurry has a positive effect on the amount of acid used to adjust a targeted pH, because microbial conversions of organic matter that increase the buffer capacity may not yet have taken place. However, alkalization technologies are based on different approaches. For phosphorus precipitation, alkalization at a very early stage is recommended to minimize the consumption of bases. To remove as much nitrogen as possible from the slurry in the form of NH_3_ so that it can be recovered in an acid reserve, the alkalization should only be carried out after the urea has been completely degraded, even though this significantly increases the consumption of bases.

## Figures and Tables

**Table 1:** Characteristics of fattening pig, dairy cow and sow slurry (fresh material) in week 0 and 8 depending on cold (4.7 ± 1.1 °C) and warm storage conditions (23.6 ± 2.1 °C).

		Fattening Pig			Dairy Cow			Sow		
Week and Storage Conditions		0	8 Cold	8 Warm	0	8 Cold	8 Warm	0	8 Cold	8 Warm
Ingredients ^1^										
Dry residue	%	8.30	8.27	7.23	9.80	10.03	8.90	2.50	2.37	2.20
N	kg m^−3^	4.81	4.92	5.02	4.20	4.22	4.11	5.37	5.41	5.03
NH_4_-N	kg m^−3^	2.88	2.99	3.17	2.42	2.37	2.51	4.97	4.20	4.37
P_2_O_5_	kg m^−3^	2.56	2.94	3.36	1.16	1.53	1.58	0.92	0.87	0.93
K_2_O	kg m^−3^	4.14	4.72	5.03	4.14	5.31	5.36	1.70	1.72	1.78
Acetic acid	g kg^−1^	7.00	5.83	2.67	6.20	5.83	6.87	2.70	2.77	2.50
Propionic acid	g kg^−1^	1.60	1.53	2.17	1.20	1.17	1.97	0.14	0.15	0.29
Acetic acid equivalent ^2^	g kg^−1^	9.30	8.10	4.90	7.70	7.27	9.03	2.90	3.00	2.93
TAN	kg N m^−3^	2.50	2.35	3.33	2.48	2.31	2.96	4.80	4.88	4.71
TIC	kg C m^−3^	1.48	1.48	1.99	1.43	1.31	1.33	1.06	1.45	1.89

^1^ Physico-chemical parameters, macronutrients and volatile fatty acids analyzed by an external independent laboratory; total ammonia nitrogen (TAN) and total inorganic carbon (TIC) were determined by own analysis; ^2^ Acetic acid equivalents were calculated from the acetic acid, propionic acid, butyric acid, iso-butyric acid, valeric acid, iso-valeric acid and n-caproic acid.

## Appendix A

**Table A1:** Additional characteristics of fattening pig, dairy cow and sow slurry (fresh material) in week 0 and 8 depending on cold (4.7 ± 1.1 °C) and warm storage conditions (23.6 ± 2.1 °C).

		Fattening Pig			Dairy Cow			Sow		
Week and Storage Conditions		0	8 Cold	8 Warm	0	8 Cold	8 Warm	0	8 Cold	8 Warm
Ingredients ^1^										
MgO	kg m^−3^	2.01	2.22	2.51	0.81	1.03	1.02	0.62	0.61	0.64
CaO	kg m^−3^	3.63	3.72	4.30	3.13	4.03	3.96	0.89	0.77	0.86
S	kg m^−3^	0.55	0.65	0.69	0.53	0.67	0.60	0.35	0.36	0.35
Cu	g m^−3^	15.00	16.10	18.47	3.50	4.48	4.47	2.87	2.75	2.93
Zn	g m^−3^	80.40	90.87	102.40	17.40	22.10	21.97	16.20	15.47	16.47
Butyric acid	g kg^−1^	0.82	0.77	<0.05	0.51	0.50	0.37	0.09	0.09	0.11
Iso-butyric acid	g kg^−1^	0.35	0.35	0.38	0.12	0.14	0.24	<0.05	0.06	0.12
Valeric acid	g kg^−1^	0.06	0.05	0.05	0.05	<0.05	0.10	<0.05	<0.05	<0.05
Iso-valeric acid	g kg^−1^	0.38	0.38	0.37	0.08	0.09	0.16	0.05	0.06	0.10
n-caproic acid	g kg^−1^	<0.05	<0.05	<0.05	<0.05	<0.05	<0.05	<0.05	<0.05	<0.05

^1^ macronutrients, micronutrients and volatile fatty acidy analyzed by external independent laboratory.

**Table A2:** Initial pH value of fattening pig, dairy cow and sow slurry as well as amount of acid under cold (4.7 ± 1.1 °C) and warm (23.6 ± 2.1 °C) storage conditions in the pH range initial pH value to 7.0, 7.0 to 5.5 and 5.5 to 3.0 over storage period of 12 weeks (means (SEM)), same letters within rows and omitted letters indicate no significant differences among the weeks.

Slurry Characteristics			Week					
**Fattening Pig**			**0**	**1**	**2**	**4**	**6**	**12**
initial pH value		cold	7.34 (0.05) ^ab^	7.31 (0.02) ^a^	7.29 (0.07) ^a^	7.54 (0.03) ^ab^	7.58 (0.03) ^b^	8.09 (0.09) ^c^
		warm	7.34 (0.05) ^a^	7.08 (0.02) ^a^	7.20 (0.03) ^a^	7.92 (0.06) ^b^	8.21 (0.13) ^bc^	8.52 (0.09) ^c^
initial to 7.0	mol HCl kg^−1^ slurry	cold	0.021 (0.004) ^ab^	0.017 (0.001) ^a^	0.018 (0.002) ^a^	0.027 (0.001) ^ab^	0.032 (0.001) ^b^	0.051 (0.005) ^c^
		warm	0.021 (0.004) ^ab^	0.005 (0.001) ^a^	0.009 (0.002) ^a^	0.037 (0.003) ^bc^	0.053 (0.007) ^c^	0.038 (0.003) ^bc^
7.0 to 5.5		cold	0.211 (0.010) ^a^	0.219 (0.005) ^ab^	0.220 (0.002) ^ab^	0.225 (0.005) ^ab^	0.225 (0.005) ^ab^	0.243 (0.004) ^b^
		warm	0.211 (0.010) ^ab^	0.202 (0.003) ^ab^	0.203 (0.004) ^ab^	0.242 (0.005) ^b^	0.244 (0.023) ^b^	0.182 (0.008) ^a^
5.5 to 3.0		*cold*	0.198 (0.005)	0.197 (0.007)	0.195 (0.005)	0.182 (0.002)	0.180 (0.002)	0.156 (0.015)
		*warm*	0.198 (0.005) ^bc^	0.245 (0.006) ^cd^	0.262 (0.011) ^d^	0.225 (0.020) ^bcd^	0.178 (0.015) ^ab^	0.132 (0.010) ^a^
**Dairy cow**								
initial pH value		cold	7.74 (0.03) ^b^	7.74 (0.03) ^b^	7.66 (0.03) ^ab^	7.47 (0.07) ^ab^	7.45 (0.05) ^a^	7.42 (0.08) ^a^
		warm	7.74 (0.03) ^bc^	7.07 (0.05) ^a^	7.14 (0.03) ^a^	7.56 (0.05) ^b^	7.93 (0.03) ^c^	8.50 (0.14) ^bc^
initial to 7.0	mol HCl kg^−1^ slurry	cold	0.029 (0.001)	0.031 (0.001)	0.030 (0.003)	0.020 (0.002)	0.021 (0.004)	0.021 (0.004)
		warm	0.029 (0.001) ^c^	0.004 (0.002) ^a^	0.007 (0.001) ^ab^	0.022 (0.002) ^bc^	0.032 (0.001) ^bc^	0.052 (0.006) ^bc^
7.0 to 5.5		cold	0.152 (0.002) ^a^	0.154 (0.002) ^a^	0.163 (0.002) ^ab^	0.171 (0.004) ^b^	0.167 (0.004) ^ab^	0.177 (0.004) ^b^
		warm	0.152 (0.002) ^ab^	0.153 (0.005) ^a^	0.154 (0.004) ^ab^	0.160 (0.005) ^ab^	0.177 (0.005) ^b^	0.170 (0.006) ^ab^
5.5 to 3.0		cold	0.193 (0.001)	0.184 (0.004)	0.187 (0.003)	0.185 (0.005)	0.193 (0.006)	0.193 (0.011)
		warm	0.193 (0.001)	0.240 (0.013)	0.243 (0.000)	0.237 (0.006)	0.222 (0.012)	0.198 (0.014)
**Sow**								
initial pH value		cold	8.89 (0.05) ^a^	9.22 (0.01) ^d^	9.12 (0.01) ^cd^	9.01 (0.01) ^bc^	8.97 (0.02) ^ab^	8.94 (0.02) ^ab^
		warm	8.89 (0.05) ^ab^	8.97 (0.02) ^bc^	8.83 (0.01) ^a^	8.90 (0.01) ^ab^	8.83 (0.02) ^a^	9.07 (0.00) ^c^
initial to 7.0	mol HCl kg^−1^ slurry	cold	0.064 (0.009) ^a^	0.152 (0.002) ^d^	0.143 (0.001) ^cd^	0.128 (0.001) ^bc^	0.125 (0.002) ^bc^	0.118 (0.002) ^b^
		warm	0.064 (0.009) ^a^	0.144 (0.002) ^c^	0.127 (0.005) ^bc^	0.120 (0.006) ^bc^	0.120 (0.006) ^bc^	0.110 (0.006) ^b^
7.0 to 5.5		cold	0.085 (0.007) ^a^	0.151 (0.001) ^b^	0.159 (0.001) ^c^	0.174 (0.000) ^d^	0.180 (0.002) ^de^	0.189 (0.001) ^e^
		warm	0.085 (0.007) ^a^	0.173 (0.004) ^c^	0.173 (0.004) ^c^	0.155 (0.003) ^c^	0.163 (0.006) ^c^	0.121 (0.006) ^b^
5.5 to 3.0		cold	0.066 (0.002) ^a^	0.081 (0.002) ^b^	0.086 (0.001) ^bc^	0.090 (0.001) ^c^	0.088 (0.001) ^bc^	0.085 (0.002) ^bc^
		warm	0.066 (0.002) ^a^	0.091 (0.001) ^b^	0.102 (0.000) ^bc^	0.104 (0.002) ^c^	0.093 (0.004) ^bc^	0.063 (0.005) ^a^
